# Non‐Viral Cytokine‐Inducible SH2 Containing Protein Locus‐Specific Integrated Fibroblast Activation Protein Alpha‐Targeting Chimeric Antigen Receptor T Cells Achieve Potent Antitumor Efficacy in Glioblastoma

**DOI:** 10.1002/mco2.70702

**Published:** 2026-03-26

**Authors:** Xin Dong, Yao Sun, Yuetong Guo, Jiao Wang, Fei Wang, Ziming Wang, Ruizhen Li, Fei Xie, Tingting Tan, Baijie Cheng, Ronghan Huang, Shu Zhang, Xiaotong Lin, Zhaoze Guo, Hubing Wu, Hao Wu, Xubiao Zhang, Guozhu Xie

**Affiliations:** ^1^ Department of Radiation Oncology Nanfang Hospital Southern Medical University Guangzhou Guangdong China; ^2^ Full Circles Therapeutics Cambridge Massachusetts USA; ^3^ Nanfang PET Center Nanfang Hospital Southern Medical University Guangzhou Guangdong China; ^4^ Department of Pathology Guangdong Sanjiu Brain Hospital Guangzhou Guangdong China; ^5^ Breast Center Department of General Surgery Nanfang Hospital Southern Medical University Guangzhou Guangdong China; ^6^ Department of Neurosurgery Guangdong Sanjiu Brain Hospital Guangzhou Guangdong China; ^7^ Guangdong Provincial Key Laboratory of Viral Hepatitis Research Guangzhou Guangdong China

**Keywords:** chimeric antigen receptor cell therapy, clustered regularly interspaced short palindromic repeat/Cas9, cytokine‐inducible SH2‐containing protein, fibroblast activation protein alpha, glioblastoma

## Abstract

Chimeric antigen receptor T (CAR‐T) cells have been used to treat patients with glioblastoma (GBM) in clinical trial settings by targeting GBM‐associated antigens. However, the efficacy of these CAR‐T cells remains limited mainly due to the heterogeneous expression of tumor antigen and their anergy in the tumor microenvironment (TME). Cytokine‐inducible SH2‐containing protein (CIS, encoded by the gene *CISH*) is a potent intracellular checkpoint inducing T‐cell anergy. Here, we identified fibroblast activation protein alpha (FAPα) as a highly attractive target for CAR‐T cell therapy against GBM based on its dual expression pattern (on tumor cells and perivascular cells) in GBM. A panel of nanobodies specific for FAPα was isolated, and FAPα‐targeting CAR‐T cells were developed using the isolated nanobody to verify their specific cytotoxicity to GBM cells. Furthermore, a non‐viral circular single‐stranded DNA (cssDNA)‐based CRISPR/Cas9‐targeted genome‐editing (cssDNA/CRISPR/Cas9) technology was used to integrate CAR cassettes at the *CISH* locus to generate *CISH*‐knockout (*CISH*‐KO) CAR‐T cells. The resulting *CISH*‐KO‐CAR‐T cells exhibited robust proliferation and potent anti‐GBM activity in vitro and in vivo. Thus, our results provide novel engineered CAR‐T cells with enhanced efficacy against GBM.

## Graphical abstract

FAPα is a dual target on glioblastoma cells and perivascular cells. Using a high‐affinity anti‐FAPα nanobody, we generated *CISH*‐KO FAPα‐CAR‐T cells via non‐viral CRISPR–Cas9 integration. These cells demonstrated enhanced proliferation and superior antitumor efficacy in patient‐derived organoids and orthotopic mouse models compared to conventional CAR‐T cells. Image created with BioRender.com with permission.

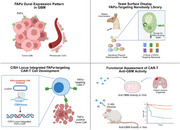

## 1 Introduction

Glioblastoma (GBM) is one of the most common and lethal malignant brain tumors in adults. Less than 10% of patients with GBM could survive for 5 years even though they had received the standard‐of‐care treatment, including surgical resection, chemotherapy, and radiotherapy [1], highlighting an urgent need for a more effective therapeutic approach.

Chimeric antigen receptor T (CAR‐T) cell therapy has been successfully developed and approved for treating malignant hematological tumors and achieved a remarkable clinical efficacy [2]. However, it remains a big challenge to develop an effective CAR‐T cell therapy against solid tumors, including GBM. Previous studies have shown limited efficacy of CAR‐T cells in treating patients with GBM by targeting GBM‐specific and associated antigens, such as EGFRvIII, HER2, IL13RA2, EphA2, or GD2, partially due to heterogeneity or loss of tumor antigens [3, 4, 5, 6]. Therefore, the rational selection of a therapeutic target is crucial for improving the efficacy of CAR‐T cells in GBM.

In addition to target selection, another important factor resulting in the failure of CAR‐T cell therapy against solid tumors is intrinsic exhaustion of T cells in the hostile tumor microenvironment, which is correlated with the upregulated expression of some immune checkpoint molecules (e.g., PD‐1, Lag‐3, CTLA‐4, TIM‐3, and TIGIT). The blockade of PD‐1 by antibodies or PD‐1 knockout (KO) has shown an increased antitumor efficacy of CAR‐T cells in some solid tumor models [7, 8]. The cytokine‐inducible SH2‐containing protein (CIS, encoded by *CISH*), a member of the suppressor of cytokine signaling (SOCS) family, was a newly identified intracellular checkpoint molecule and showed a key role in suppressing the ability of T cells to recognize and kill tumor cells [9]. Therefore, CIS has been considered to be a potent therapeutic checkpoint molecule for adoptive cell therapy.

Notably, current CAR‐T products approved by the FDA are prepared by engineering T cells using lentivirus‐mediated gene transduction technology. However, the use of a virus in CAR‐T cell production becomes one area of concern because of the disadvantages of the increased risk of tumorigenicity due to random insertional mutagenesis [10, 11, 12], high costs [13], and host‐specific immune responses to virus‐derived DNA [14, 15]. To address these defects, CRISPR–Cas9 gene editing technology was used to knock‐in CAR cassettes at a specific genomic locus, generating CAR‐T cells with PD‐1‐KO or universal CAR‐T cells with T‐cell receptor α constant (TRAC)‐KO in preclinical animal models and clinical trials [16, 17]. CRISPR–Cas9‐mediated targeted CAR insertion traditionally uses a homology‐directed repair (HDR) DNA donor template encapsulated in recombinant associated adenovirus (rAAV). However, limited editable fragment lengths and potential viral‐related safety restricted the application of rAAV as a donor template payload. Currently, non‐viral DNA donor templates represent an innovative tool for CRISPR–Cas9‐targeted genome editing, which primarily relies on circular or linear double‐stranded DNA (dsDNA) and single‐stranded DNA (ssDNA) donors to achieve HDR at a locus of interest with double‐strand break (DSB). It is notable, however, that dsDNA and ssDNA have been previously demonstrated to have high toxicity to immune cells, low knock‐in efficiency, or inefficient production [18, 19]. In contrast, circular single‐stranded DNA (cssDNA) is characterized by its high stability and low cytotoxicity, and has been recently shown to be a superior homology‐directed repair donor template for efficient targeted genetic knock‐in in primary immune cells [20].

In this study, we first identified fibroblast activation protein alpha (FAPα) as a highly attractive target for CAR‐T cell therapy against GBM based on its dual expression pattern (on tumor cells and perivascular cells) in GBM. A panel of nanobodies, also known as camelid VHHs (variable heavy chain domains of heavy chain antibody) [21], specific for FAPα was isolated using a yeast surface display library, and FAPα‐targeting CAR‐T cells were developed and verified for their specific cytotoxicity to GBM cells. Furthermore, a non‐viral cssDNA‐based CRISPR/Cas9‐targeted genome‐editing (cssDNA/CRISPR/Cas9) technology was used to integrate CAR cassettes at the *CISH* locus to generate *CISH*‐KO‐CAR‐T cells. The resulting *CISH*‐KO‐CAR‐T cells exhibited robust proliferation and potent anti‐GBM activity in vitro and in vivo. Our proof‐of‐concept study demonstrates that *CISH*‐KO FAPα‐targeting CAR‐T cells engineered by non‐viral *CISH* locus‐specific integration represent a promising therapeutic approach for the treatment of GBM.

## 2 Results

### 2.1 High Expression of FAPα on Both Tumor Cells and Their Supporting Vascular Network in GBM

**FIGURE 1:**
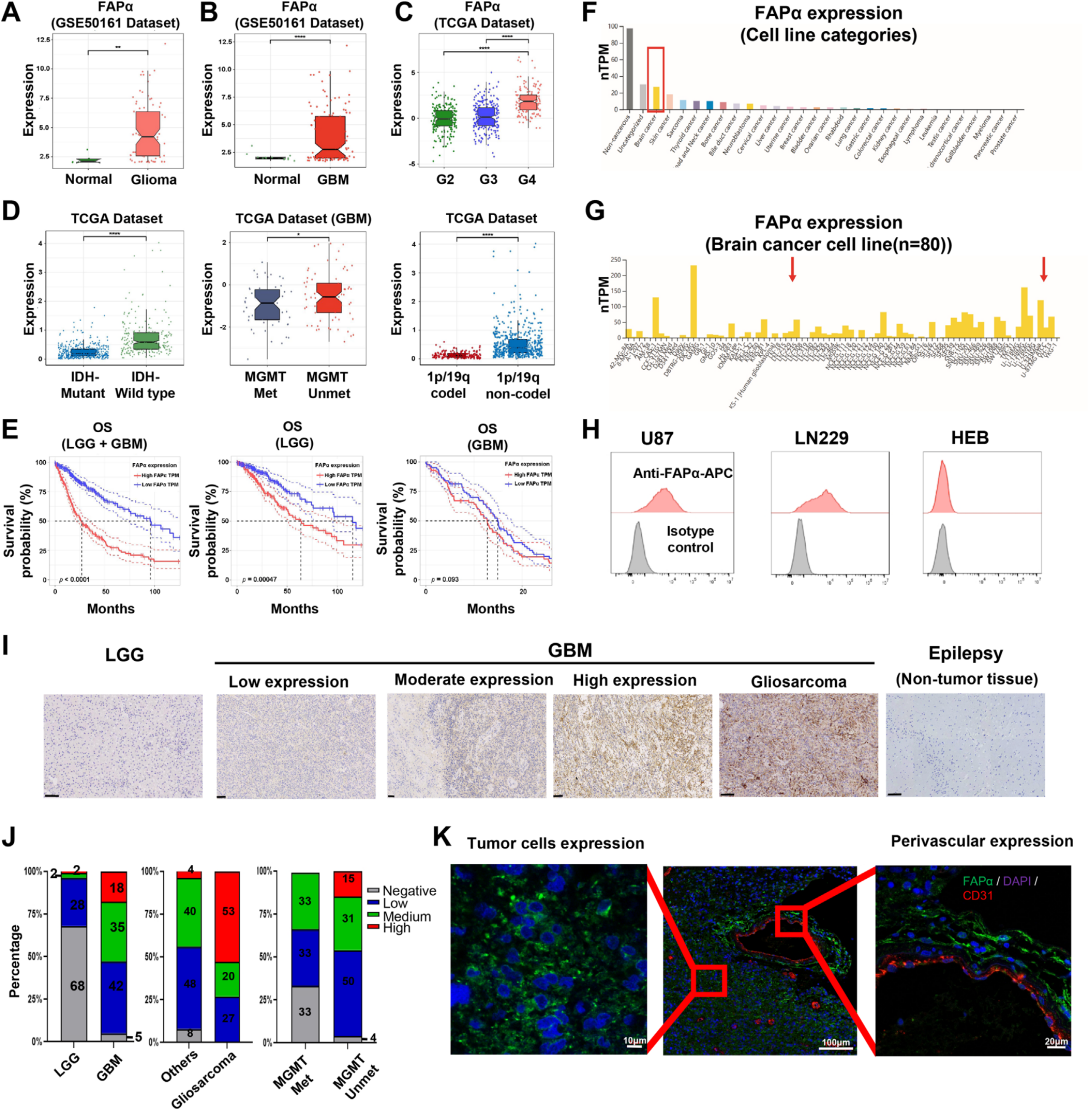
Expression of FAPα in glioblastoma (GBM). (A) The mRNA expression of FAPα between normal and glioma tissues. Analysis of GSE50161 dataset (*n* = 117 for gliomas and *n* = 13 for normal). **p < 0.01 (*t*‐test). (B) The mRNA expression of FAPα between normal and GBM tissues. ****p < 0.0001 (*t*‐test). (C) FAPα expression increased with higher pathological grades in all gliomas. ****p < 0.0001 (pairwise comparisons of Tukey's Honest Significant Difference). (D) Analysis of the relationship between FAPα and clinical characteristics of glioma in the TCGA database. *p < 0.05, ****p < 0.0001 (*t*‐test). (E) Kaplan–Meier analysis of overall survival analysis of FAPα in glioma patients in the TCGA dataset. (F and G) The RNA sequencing data from the Human Protein Atlas (HPA) database (version 23.0); red arrow indicating the expression of FAPα in LN229 and U87 GBM cell lines. (H) FAPα expression on the surface of GBM (U87 and LN229) and normal human glial cell lines (HEB) detected by flow cytometry. (I) The in situ expression of FAPα in patient‐derived glioma tissues from low‐grade glioma (LGG) patients (*n* = 40) and GBM patients (*n* = 55, including 16 gliosarcoma patients), and normal brain tissue from epilepsy patients (*n* = 6) determined by immunohistochemistry. Scale bar: 100 µm. (J) The quantitative analysis of FAPα expression in patient‐derived glioma tissues; numbers in the histogram indicate percentages. (K) The specific location of FAPα expression in GBM tissues detected by immunofluorescence assay.

FAPα is a Type II transmembrane serine protease, extensively expressed on cancer‐associated fibroblasts (CAFs) in solid tumors and almost undetectable in normal tissues, making it an ideal marker for targeting tumors [22]. Interestingly, previous studies found that FAPα was significantly expressed in GBM but not in normal brain tissue [23, 24], even though CAFs rarely existed in GBM [23, 25]. To demonstrate whether FAPα is an ideal antigen for CAR‐T cells against GBM, the gene expression profile data for glioma patients were obtained from GSE50161 and TCGA databases, and analyzed for mRNA expression of FAPα. The results showed that both glioma and GBM had a significant overexpression of FAPα compared to normal brain tissue (Figure 1A,B), and its expression increased with higher pathological grades in all gliomas (Figure 1C). Additionally, higher FAPα expression was also correlated with IDH wild‐type status, unmethylated MGMT, and the absence of 1p/19q co‐deletion (Figure 1D), which were important indicators of poor clinical prognosis for GBM [26]. To determine whether elevated FAPα expression was correlated to poorer prognosis, overall survival (OS) was compared between patients with high (above the mean level) and low (below the mean level) FAPα expression. The results showed that FAPα‐high patients had worse OS than FAPα‐low patients in all grades of glioma, including low‐grade glioma (LGG) and GBM (Figure 1E). To demonstrate whether FAPα was expressed on tumor cells, we analyzed the RNA sequencing data from the Human Protein Atlas (HPA) database (version 23.0), showing that FAPα mRNA was notably enriched in brain tumor cell lines (Figure 1F, G). To verify these findings at the protein level, we further analyzed FAPα expression on the surface of GBM (U87‐MG and LN229) and normal human glial (HEB) cell lines by flow cytometry. In line with mRNA findings, FAPα was highly expressed on the surface of the tested GBM cell lines, but undetectable in the normal human glial cell line (Figure 1H).

Next, we detected the in situ expression of FAPα in patient‐derived glioma tissues from 40 LGG patients and 55 GBM patients, including 16 gliosarcoma patients, and normal brain tissue from six epilepsy patients by immunohistochemical (IHC) analyses (Figure 1I, J). The results showed that the FAPα expression was significantly higher in GBM than in LGG, and undetectable in all normal brain tissues from epilepsy patients (Figure 1I, J). Notably, gliosarcoma, a more aggressive variant of GBM, exhibited remarkably high expression compared to other GBM (Figure 1I, J). Additionally, the high expression of FAPα was also observed in GBM with an unmethylated MGMT promoter compared with those with a methylated MGMT promoter, indicating potential resistance to temozolomide (Figure 1J). Immunofluorescence (IF) was further used to clarify the specific location of FAPα expression in GBM tissues. As shown in Figure 1K, FAPα was not only highly expressed in the main tumor parenchyma (tumor cells), but also in their perivascular cells. However, FAPα was not expressed in CD31‐positive vascular endothelial cells, which was consistent with previous studies indicating FAPα overexpression on vascular pericytes supporting tumor vessels [27, 28]. This dual expression pattern of FAPα on both tumor cells and perivascular cells in GBM suggests that FAPα is a highly attractive target for CAR‐T cell therapy against GBM.

### 2.2 Isolation of Nanobodies Specific for FAPα Using a Yeast Surface Display Library From a rhFAPα‐Immunized Alpaca

**FIGURE 2:**
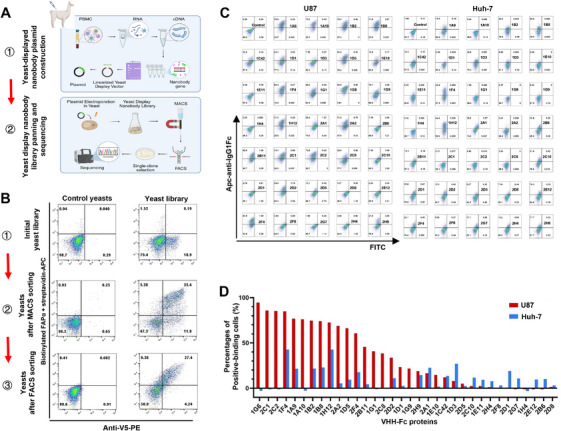
Isolation of nanobodies specific for FAPα using a yeast surface display library. (A) Schematic diagram for selecting high‐affinity nanobodies specific for FAPα by using a yeast surface display library. Image created with BioRender.com with permission. (B) A pool of yeast cells that could bind to biotin‐labeled FAPα protein is selected after magnetic‐activated cell sorting and subsequent fluorescence‐activated cell sorting for positive selection, as detected by flow cytometry; anti‐V5 tag antibody is used to detect nanobody protein on yeast cells, and biotinylated FAPα and streptavidin‐APC are used to detect the binding of FAPα to nanobody on yeast cells. (C) VHH‐Fc fusion protein is used to verify their binding to FAPα on U87 cells (a FAPα‐positive GBM cell line) and Huh‐7 cells (a hepatocellular carcinoma cell line with high CD26 expression but no FAPα) by flow cytometry. (D) The percentages of positive‐binding cells.

Isolation of antibodies specific for FAPα is the basis for developing GBM‐specific CAR‐T therapy. We used a yeast surface display library from a recombinant human FAPα protein (rhFAPα)‐immunized alpaca to identify high‐affinity nanobodies (also known as VHHs) specific for FAPα (Figure 2A). After magnetic‐activated cell sorting (MACS) and subsequent fluorescence‐activated cell sorting (FACS) for positive selection (Figure 2B), a pool of yeast cells that could bind to biotinylated FAPα protein was selected, as detected by flow cytometry in which anti‐V5 tag antibody was used to detect VHH protein on yeast cells, and biotinylated FAPα and streptavidin‐APC were used to detect the binding of FAPα to VHH on yeast cells.

A total of 117 single yeast clones were randomly picked, and their bindings to biotinylated FAPα protein were then confirmed by flow cytometry (Figure S1). The VHH sequence for each clone was determined by Sanger sequencing of the PCR products from each positive‐binding yeast clone. Eventually, 40 different VHH sequences were identified. To further determine the binding specificity of VHH to FAPα expressed on tumor cells, we produced VHH‐Fc fusion protein using a mammalian expression system by transiently transfecting VHH‐Fc‐overexpressing plasmid DNA into HEK293F cells, and then used flow cytometry to verify their binding to FAPα on 293T cells with FAPα overexpression (FAPα‐293T cells), wild‐type 293T (wt‐293T) cells (Figure S2), and U87 cells (a FAPα‐positive GBM cell line) (Figure 2C). The results showed that 23 of 40 VHH‐Fc proteins could positively bind to both U87 and FAPα‐293T but not wt293T cells. A previous study has shown that CD26, a membrane‐bound protein with dipeptidyl peptidase IV activity that was widely expressed in normal tissues, had a highly similar structure to FAPα, with 48% amino acid sequence identity [29]. In order to avoid the binding of the nanobodies to CD26, Huh‐7 cells, a hepatocellular carcinoma cell line that highly expressed CD26 but not FAPα, were used as a negative selection condition (Figure 2C). As expected, among 23 VHH‐Fc proteins that could bind to U87 cells, 11 could also bind to FAPα^−^ CD26^+^ Huh‐7 cells (Figure 2C, D), indicating the necessity of CD26‐positive cells for negative selection.

Based on these screening outcomes, we successfully identified 12 nanobodies that specifically bound to human FAPα, which provided important candidate FAPα‐binding domains for CAR‐T cells.

### 2.3 Screening CAR‐T Cells That Specifically Kill FAPα^+^ GBM Cells

**FIGURE 3:**
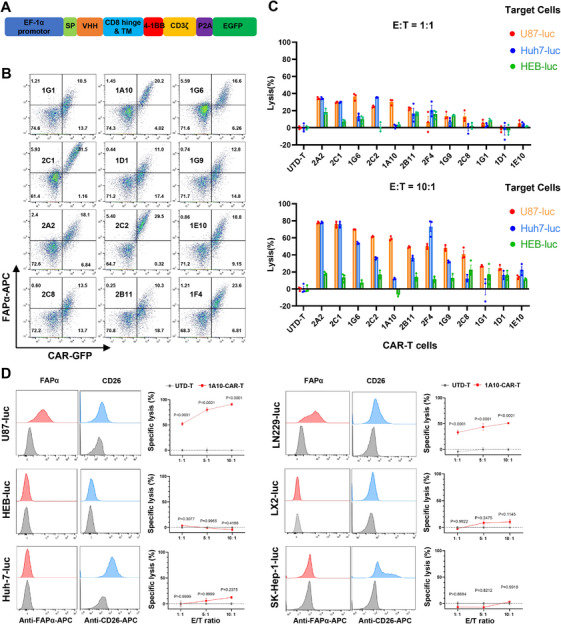
Screening CAR‐T cells that specifically kill FAPα^+^ GBM cells. (A) CAR construct containing a VHH, a CD8α hinge and transmembrane domain, a 4‐1BB co‐stimulatory domain and a CD3ζ activation domain, followed by self‐cleavage P2A and the enhanced green fluorescent protein (EGFP). (B) The transduced T cells are analyzed for GFP and CAR expression by staining with biotinylated FAPα antigen and streptavidin labeled with APC. (C) The cytotoxicity of CAR‐T cells against luciferase‐overexpressing FAPα^+^ U87 (U87‐luc) cells, CD26^+^ Huh‐7 (Huh‐7‐luc) cells, and FAPα^−^CD26^−^ HEB cells (HEB‐luc). (D) The cytotoxicity of 1A10‐CAR‐T against multiple cell lines with different expression patterns of FAPα and CD26. Data are presented as the mean ± SEM (*n* = 3 independent experiments).

To identify CAR‐T cells that specifically kill FAPα^+^ GBM cells, we constructed 12 FAPα‐targeting CARs by cloning the sequences of the identified VHHs into a CAR backbone containing a CD8α hinge and transmembrane domain, a 4‐1BB co‐stimulatory domain, and a CD3ζ activation domain, followed by self‐cleavage P2A and the enhanced green fluorescent protein (EGFP) [30] (Figure 3A). Human CD3^+^ T cells were purified from donor peripheral blood mononuclear cells (PBMCs), activated with polymeric nanomatrix conjugated to humanized CD3 and CD28 agonist for 2 days, and then transduced with lentiviruses to express FAPα CAR. Three days later, the transduced T cells were analyzed for GFP and CAR expression by staining with biotinylated FAPα antigen and streptavidin labeled with APC. As shown in Figure 3B, a panel of CAR‐T cells was generated, with 20%–40% of positive cells for GFP.

To detect the specific cytotoxicity of anti‐FAPα VHH‐CAR‐T cells, these CAR‐T cells were co‐cultured with luciferase‐overexpressing FAPα^+^ U87 (U87‐luc) cells, CD26^+^ Huh‐7 (Huh‐7‐luc) cells, and FAPα^−^CD26^−^ HEB (HEB‐luc) cells, at different effector‐to‐target (*E*:*T*) ratios for 16 h. Before co‐culture, the positive rates of all CAR‐T cells were adjusted to 20% using untransduced T cells to facilitate inter‐group comparisons. The results showed that different VHH‐CAR‐T cells exhibited variable cytotoxic effects on U87 cells (Figure 3C). Among them, 2A2, 2C1, 1G6, 2C2, 2B11, 2F4, and 1G9‐CAR‐T cells displayed strong killing capacity against U87 cells; however, they also showed notable cytotoxicity against CD26^+^ Huh‐7 cells. The 1A10‐CAR‐T cells exhibited potent cytotoxicity against FAPα^+^ U87 cells but not CD26^+^ Huh‐7 cells, regardless of effector‐to‐target ratios. To further verify the specific cytotoxicity of 1A10‐CAR‐T against FAPα‐positive cells, we detected the cytotoxicity of 1A10‐CAR‐T using multiple cell lines with different expression patterns of FAPα and CD26 at 1:1, 5:1, and 10:1 of *E*:*T* ratios (Figure 3D). The results demonstrated a significant specific cytotoxicity of 1A10‐CAR‐T against FAPα‐positive cells but not CD26‐positive cells, guiding our selection of 1A10‐CAR‐T as the ideal CAR‐T cell candidate for further experiments.

### 2.4 Efficient CAR‐T Cell Engineering by Non‐Viral cssDNA‐Based CRISPR/Cas9‐Targeted Genome Integration at *CISH* Locus

**FIGURE 4:**
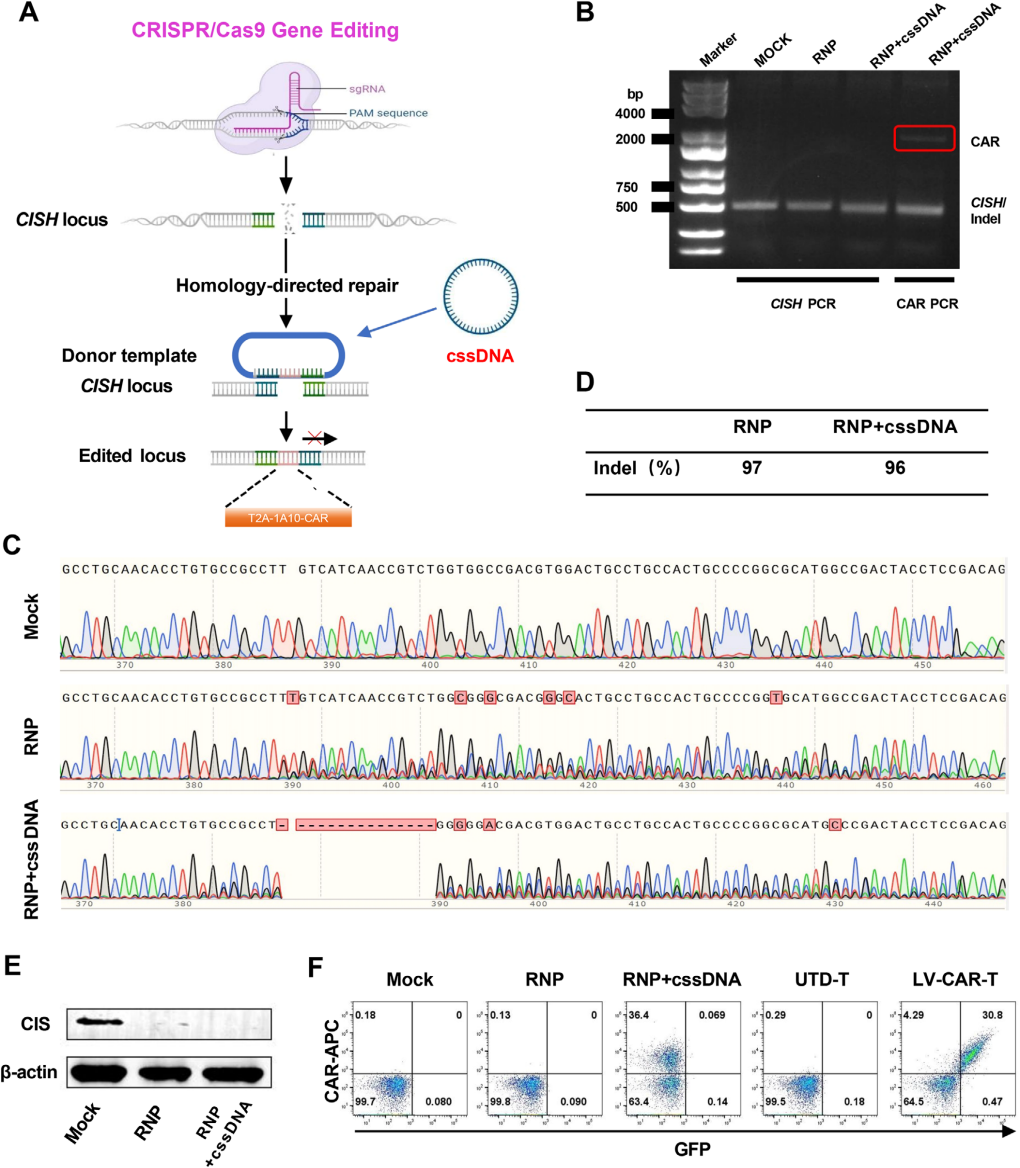
Efficient CAR‐T cell engineering by non‐viral cssDNA‐based CRISPR/Cas9‐targeted genome integration at the *CISH* locus. (A) Generation of non‐viral *CISH* locus‐specific integrated CAR‐T cells using cssDNA HDR donor templates targeting the *CISH* locus. Image created with BioRender.com with permission. (B) The DNA fragment of CAR was amplified using a pair of primers complementary to the *CISH* DNA. (C) Chromatograms showing sequencing data of PCR products of *CISH* from Mock‐T, RNP‐T, or CISH‐KO‐CAR‐T cells. (D) The indel percentage is measured by Inference of CRISPR Edits (ICE) analysis. (E) Western blot of CIS protein. (F) The percentages of CAR‐positive T cells are detected by flow cytometry. UTD‐T, untransduced‐T cells; LV‐CAR‐T, lentivirus‐transduced CAR‐T.

CIS (encoded by *CISH*) has been shown to be a potent intracellular checkpoint inducing T‐cell anergy [31, 32]. To enhance the anti‐GBM effect of CAR‐T cells, we next applied a non‐viral cssDNA‐based CRISPR/Cas9‐targeted genome integration technology to generate *CISH*‐knockout FAPα‐targeting CAR‐T cells (Figure 4A).

*CISH*‐specific cssDNA donor template and a ribonucleoprotein (RNP) complex consisting of Cas9 protein and a single guide RNA (sgRNA) targeting the *CISH* locus were delivered into CD3^+^ T cells by electroporation (EP). Genomic DNA was purified, and fragments containing indel sites were amplified by PCR. As shown in Figure 4B, DNA fragment of CAR could be amplified when using a pair of primers complementary to *CISH* DNA, indicating that the CAR fragment was integrated at the *CISH* locus. DNA sequencing was further carried out (Figure 4C), and the indel percentages were measured by Inference of CRISPR Edits (ICE) analysis. The results showed that the indel percentages in RNP and RNP combined with cssDNA (RNP+cssDNA) groups were 96% and 97%, respectively (Figure 4D), indicating high knockout rates of *CISH*, which could be confirmed by a Western blot of CIS protein (Figure 4E). Notably, 36.4% of CAR‐positive T cells were detected by flow cytometry in the RNP+cssDNA group (Figure 4F), indicating highly efficient CAR knock‐in at the *CISH* locus by non‐viral cssDNA‐based CRISPR/Cas9‐targeted genome integration.

### 2.5 Non‐Viral *CISH* Locus‐Specific Integrated FAPα CAR‐T Cells Achieve Potent Anti‐GBM Efficacy In Vitro

**FIGURE 5:**
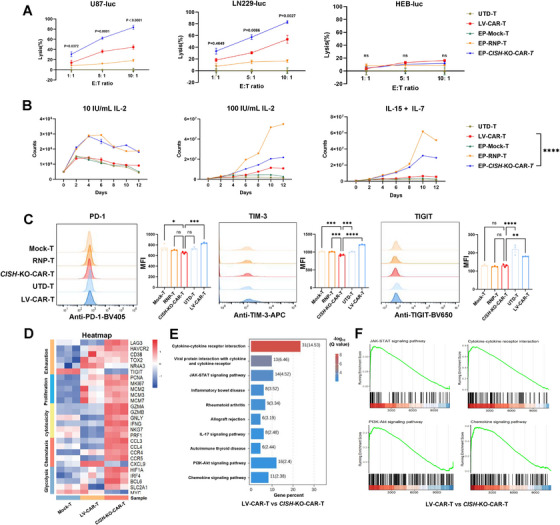
Non‐viral *CISH* locus‐specific integrated FAPα CAR‐T cells achieve potent anti‐GBM efficacy in vitro. (A) The cytotoxicity of CAR‐T cells against GBM cell lines is evaluated by co‐culturing T cells with FAPα‐positive GBM cells (U87‐luc and LN229‐luc) or FAPα‐negative normal human glial cells (HEB‐luc) for 16 h, and then detecting luciferase activity of these target cells. The percentages of specific lysis of tumor cells at different effector‐to‐target ratios (*E*:*T* ratios) are calculated. Data are presented as the mean ± SEM (*n* = 3 independent experiments). ns: no significance; E:T ratio, effector‐to‐target cell ratio. (B) *CISH*‐KO remarkably increased the proliferation ability of T cells in media with both low (10 IU/mL) and high (100 IU/mL) concentrations of IL‐2, or the mixture of IL‐15 (5 ng/mL) and IL‐7 (10 ng/mL), compared with MOCK‐T, UTD‐T, or LV‐transduced CAR‐T cells. Data are presented as the mean ± SEM (*n* = 3 independent experiments). ****p < 0.0001. (C) The expression of PD‐1, TIM‐3, and TIGIT is detected by flow cytometry, and the mean fluorescence intensity (MFI) is compared among different T cells. Data are presented as the mean ± SEM (*n* = 3 independent experiments). Each symbol represents the result of one independent experiment. ns: no significance, *p < 0.05, **p < 0.01, ***p < 0.001, ****p < 0.0001. UTD‐T, untransduced T cells; LV‐CAR‐T, lentivirus‐generated CAR‐T. (D) The gene expression profiles of T cells are determined through RNA sequencing (RNA‐Seq), and show that *CISH*‐KO‐CAR‐T significantly upregulated the expression of proliferation‐, cytotoxicity‐, chemotaxis‐, and glycolysis‐related genes. (E and F) The KEGG pathway enrichment analysis of the differentially expressed genes (DEGs) indicates that compared with LV‐CAR‐T cells, *CISH*‐KO‐CAR‐T cells are mainly enriched with pathways related to cytokine–cytokine receptor interaction, proliferation, inflammatory response, and chemokine signaling.

To determine the in vitro anti‐GBM potency of *CISH* knockout CAR‐T (*CISH*‐KO‐CAR‐T) cells, the resulting CAR‐T cells were co‐cultured with FAPα‐positive GBM cells (U87 and LN229), or FAPα‐negative normal human glial cells (HEB). We found that *CISH*‐KO‐CAR‐T cells exhibited significantly higher cytotoxicity against GBM cell lines than mock and *CISH*‐KO alone (RNP) cells (Figure 5A). More importantly, *CISH*‐KO‐CAR‐T cells had notably more potent cytotoxicity than conventional lentivirus‐transduced CAR‐T (LV‐CAR‐T) cells at all *E*:*T* ratios (Figure 5A). Additionally, *CISH*‐KO, regardless of CAR integration, remarkably increased the proliferation ability of T cells in media with both low (10 IU/mL) and high (100 IU/mL) concentrations of IL‐2, or the mixture of IL‐15 (5 ng/mL) and IL‐7 (10 ng/mL), compared with mock, untransduced (UTD‐T) or LV‐CAR‐T cells (Figure 5B), indicating the deletion of *CISH* increased the sensitivity of T cells to cytokine‐driven proliferation. To demonstrate whether the proliferation advantage of *CISH*‐KO T cells contributed to reduced exhaustion, the expression of multiple checkpoint receptors associated with T‐cell exhaustion, including PD‐1, TIM‐3, and TIGIT, was detected by flow cytometry. As shown in Figure 5C, *CISH*‐KO‐CAR‐T cells showed a significantly decreased expression of TIM‐3 and TIGIT compared with LV‐CAR‐T cells. However, *CISH‐*KO just slightly reduced the expression of PD‐1 and TIGIT compared with mock cells, indicating the increased proliferation by *CISH*‐KO was probably not due to a reduced cell exhaustion.

To further assess the impact of *CISH*‐KO on CAR‐T cells, gene expression profiles of T cells were determined through RNA sequencing (RNA‐Seq). The results showed that *CISH*‐KO‐CAR‐T cells significantly upregulated the expression of proliferation‐, cytotoxicity‐, chemotaxis‐, and glycolysis‐related genes (Figure 5D). The KEGG pathway enrichment analysis of the differentially expressed genes (DEGs) indicated that *CISH*‐KO‐CAR‐T cells were mainly enriched with pathways related to cytokine–cytokine receptor interaction, proliferation, inflammatory response, and chemokine signaling, compared with LV‐CAR‐T cells (Figure 5E, F), which was consistent with the results above showing more potent functions of *CISH*‐KO‐CAR‐T than conventional LV‐CAR‐T cells. Taken together, these results suggest that non‐viral *CISH* locus‐specific integrated CAR‐T cells have the potential to effectively eliminate GBM tumor cells compared to conventional LV‐CAR‐T cells.

### 2.6 *CISH* Locus‐Specific Integrated FAPα CAR‐T Cells Achieve Potent Anti‐GBM Efficacy in an Orthotopic GBM Xenograft Mouse Model

**FIGURE 6:**
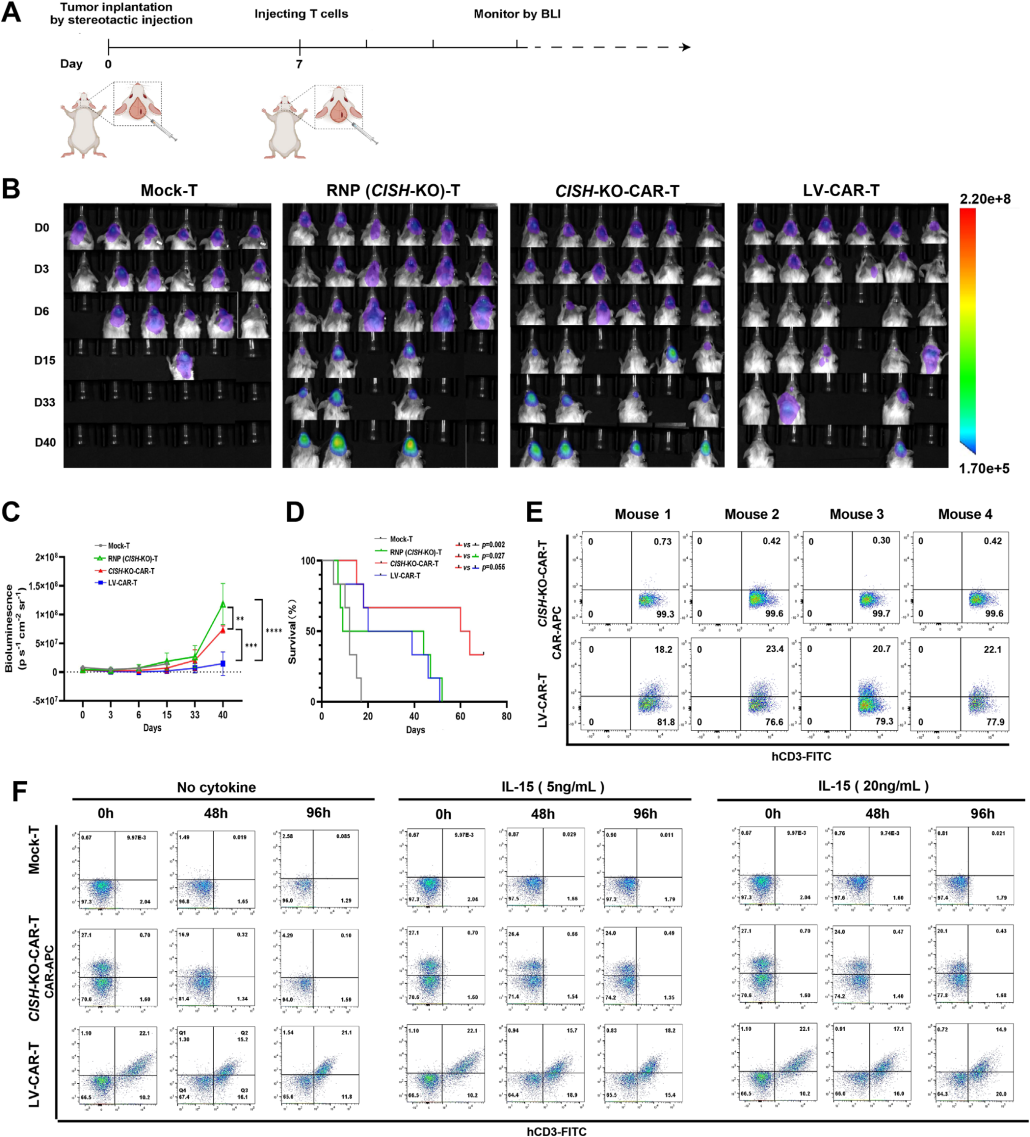
*CISH* locus‐specific integrated FAPα CAR‐T cells achieve potent anti‐GBM efficacy in an orthotopic GBM xenograft mouse model. (A) Schematic of the experimental process. Luciferase‐expressing U87 cells (3 × 10^5^ per mouse) are implanted into the right cerebral hemisphere of NCG mice by stereotactic injection. After confirmation of engraftment by bioluminescence imaging (BLI) 7 days post‐injection, mice are given a single injection of mock‐T, RNP(*CISH*‐KO)‐T, *CISH*‐KO‐CAR‐T, or LV‐CAR‐T cells (1 × 10^6^ cells per mouse) by orthotopic injection at tumor sites. Image created with BioRender.com with permission. (B) Comparison of the U87 GBM burden by BLI between mice treated with different T cells or PBS at the indicated days post‐T‐cell injection (*n* = 6). The scales for imaging are shown to the right. (C) Comparison of the total flux (luciferase signals from U87 cells) in the mice (*n* = 6) from (B). **p < 0.01; ***p < 0.001; ****p < 0.0001. (D) Kaplan–Meier survival curves (*n* = 6) of mice treated with different T cells or PBS. (E) U87 tumor tissues are isolated from mice (*n* = 4), treated with *CISH*‐KO‐CAR‐T or LV‐CAR‐T cells for 10 days, and then CAR expression of intratumoral T cells is analyzed by flow cytometry. (F) CAR expression of *CISH*‐KO‐CAR‐T and LV‐CAR‐T cells is analyzed by flow cytometry when they are cultured in media without any cytokines or with low (5 ng/mL) or high (20 ng/mL) concentration of IL‐15.

To evaluate the anti‐GBM activity of *CISH*‐KO‐CAR‐T cells in vivo, we assessed their efficacy in an orthotopic GBM xenograft mouse model. Luciferase‐expressing U87 cells (3 × 10^5^ per mouse) were implanted into the right cerebral hemisphere of NCG (NOD/ShiltJGpt‐Prkdc^−/−^IL2Rg^−/−^) mice by stereotactic injection. After confirmation of successful engraftment by bioluminescence imaging (BLI) 7 days post‐injection, mice were given a single injection of mock‐T, RNP (*CISH*‐KO)‐T, *CISH*‐KO‐CAR‐T, or LV‐CAR‐T cells (1 × 10^6^ cells per mouse) by orthotopic injection at tumor sites (Figure 6A). As shown in Figure 6B, most of the mice receiving mock‐T cells died at 16 days after T‐cell injection. *CISH*‐KO‐CAR‐T cells greatly reduced tumor burden and significantly prolonged the survival time of tumor‐bearing mice, compared with mock‐T and RNP‐T cells (Figure 6B–D). Notably, inconsistent with in vitro anti‐GBM activity, *CISH*‐KO‐CAR‐T cells did not exhibit the advantages in eliminating GBM tumors in vivo compared with LV‐CAR‐T cells (Figure 6B, C); however, the prolonged survival was still observed in mice receiving *CISH*‐KO‐CAR‐T cells (Figure 6D). Given that the expression of CAR in T cells depends on the activity of the *CISH* promoter, which is regulated by some critical cytokines including IL‐2, IL‐15, and IL‐7 [33], we speculated that CAR expression in *CISH*‐KO‐CAR‐T cells might be lost due to the lack of these cytokines in tumor‐bearing mice. To verify this hypothesis, U87 tumor tissues were isolated from mice treated with *CISH*‐KO‐CAR‐T or LV‐CAR‐T cells for 10 days, and then CAR expression of intratumoral human T cells was analyzed by flow cytometry. As expected, CAR expression was undetectable in intratumoral *CISH*‐KO‐CAR‐T cells, while LV‐CAR‐T cells could maintain a similar percentage of CAR‐positive cells in tumors (Figure 6E). To further clarify the effect of cytokines on CAR expression of *CISH*‐KO‐CAR‐T cells, we assessed CAR expression of *CISH*‐KO‐CAR‐T cells when cultured in cytokine‐free media for varying time points. As shown in Figure 6F, *CISH*‐KO‐CAR‐T cells almost lost CAR expression when cultured in cytokine‐free media for 96 h, while LV‐CAR‐T cells maintained similar levels of CAR expression. On the other hand, *CISH*‐KO‐CAR‐T cells could maintain similar levels of CAR expression as LV‐CAR‐T cells when cultured in media containing low concentration of IL‐15 (Figure 6F). These results, to some extent, explain why a superior anti‐GBM efficacy was not observed for *CISH*‐KO‐CAR‐T cells compared to LV‐CAR‐T cells in our GBM xenograft mouse model.

**FIGURE 7:**
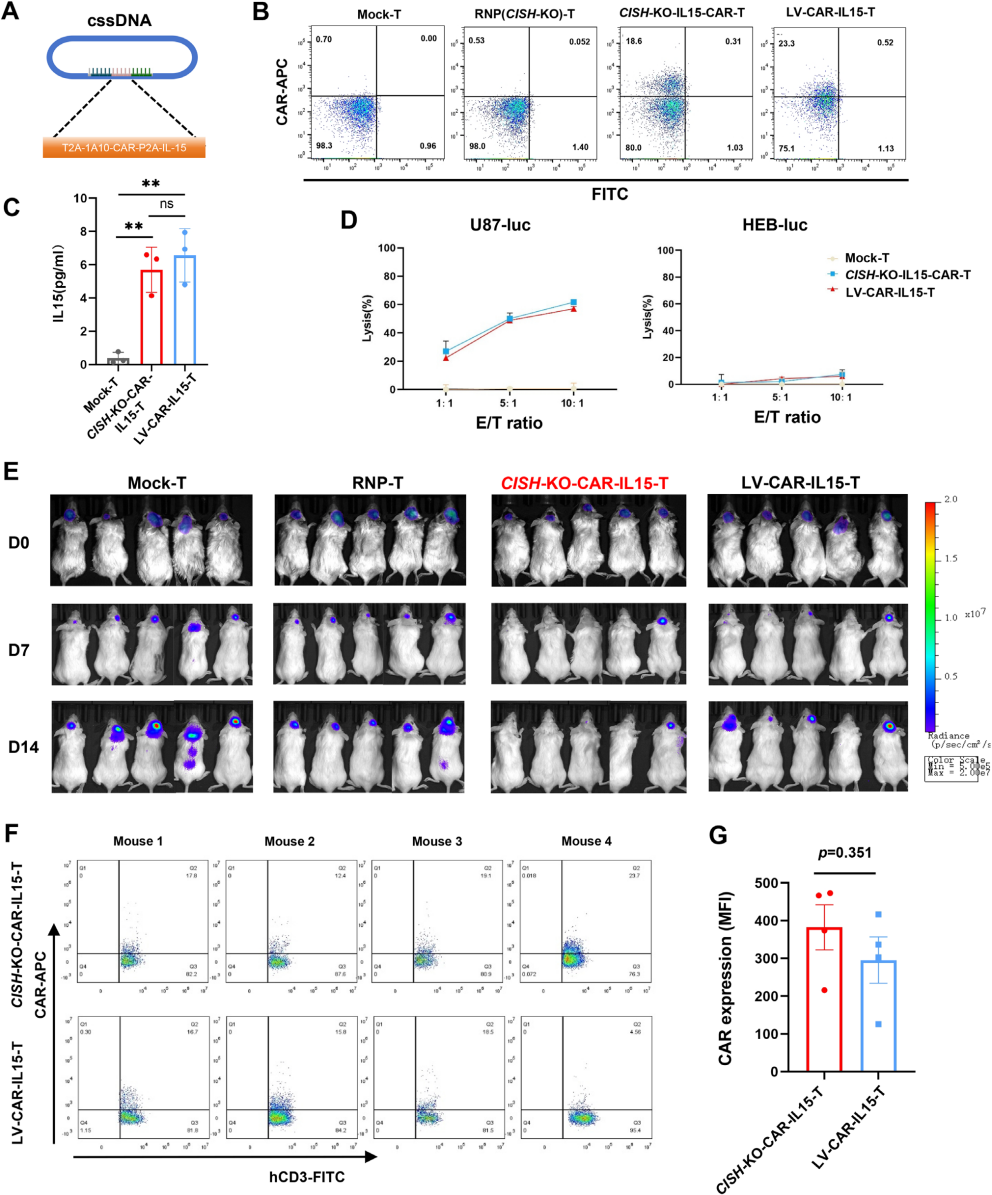
The comparison of antitumor activities between IL‐15‐expressing *CISH*‐KO‐CAR‐T cells (*CISH*‐KO‐CAR‐IL15‐T) and LV‐CAR‐T cells (LV‐CAR‐IL15‐T). (A) Schematic of a cssDNA donor template containing CAR and IL‐15. (B) The percentages of CAR‐positive T cells are detected by flow cytometry. (C) IL‐15 secretion is detected by ELISA, and no difference in secretion ability is observed between *CISH*‐KO‐ and LV‐CAR‐IL15‐T cells. Data are presented as the mean ± SEM (*n* = 3 independent experiments). ns: no significance, **p < 0.01; ***p < 0.001; ****p < 0.0001. (D) Specific lysis against FAPα^+^ U87 (U87‐luc) and FAPα^−^ HEB (HEB‐luc) cells is confirmed by luciferase assay in vitro. Data are presented as the mean ± SEM (*n* = 3 independent experiments). (E) Orthotopic GBM xenografts within the mouse brain are established using U87 cells, different CAR‐T cells are orthotopically injected at tumor sites, and tumor burden is monitored by BLI. (F) A single‐cell suspension is prepared from U87 tumors treated with *CISH*‐KO‐CAR‐IL15‐T or LV‐CAR‐IL15‐T cells for 14 days, and then CAR expression of intratumoral human T cells is analyzed by flow cytometry. (G) The mean fluorescence intensity (MFI) CAR expression is compared between *CISH*‐KO‐CAR‐IL15‐T and LV‐CAR‐IL15‐T (*n* = 4).

To further verify whether *CISH*‐KO‐CAR‐T cells had potent anti‐GBM activity compared to LV‐CAR‐T cells in vivo, we next generated *CISH*‐KO‐CAR‐T cells co‐expressing IL‐15 (*CISH*‐KO‐CAR‐IL15‐T) using a cssDNA donor template and LV‐CAR‐T cells co‐expressing IL‐15 (LV‐CAR‐IL15‐T) by lentivirus transduction (Figure 7A, B). IL‐15 secretion was detected by ELISA, and no significant difference in secretion ability was observed between *CISH*‐KO‐ and LV‐CAR‐IL15‐T cells (Figure 7C). Specific lysis against U87 cells was confirmed by luciferase assay in vitro (Figure 7D). Orthotopic GBM xenografts within mouse brain were established using U87 cells, and different IL‐15‐expressing CAR‐T cells were orthotopically injected at tumor sites. As expected, *CISH*‐KO‐CAR‐IL15‐T cells exhibited remarkably potent anti‐GBM ability compared with LV‐CAR‐IL15‐T cells (Figure 7E), indicating *CISH*‐KO boosts anti‐GBM efficacy of FAPα‐targeting CAR‐T cells in vivo. Mice were sacrificed on Day 14, and a single‐cell suspension was prepared from U87 tumor tissues. CAR expression of intratumoral human T cells was then analyzed by flow cytometry. The result showed that *CISH*‐KO‐CAR‐IL15‐T could maintain CAR expression in vivo as LV‐CAR‐IL15‐T cells (Figure 7F, G), indicating co‐expression of IL‐15 could maintain CAR expression of *CISH*‐KO‐CAR‐T cells in vivo and enhance their antitumor effects.

### 2.7 *CISH* Site‐Specific Integrated FAPα CAR‐T Cells Effectively Kill Primary Human GBM Cells in a Patient‐Derived Glioblastoma Organoid Model

**FIGURE 8:**
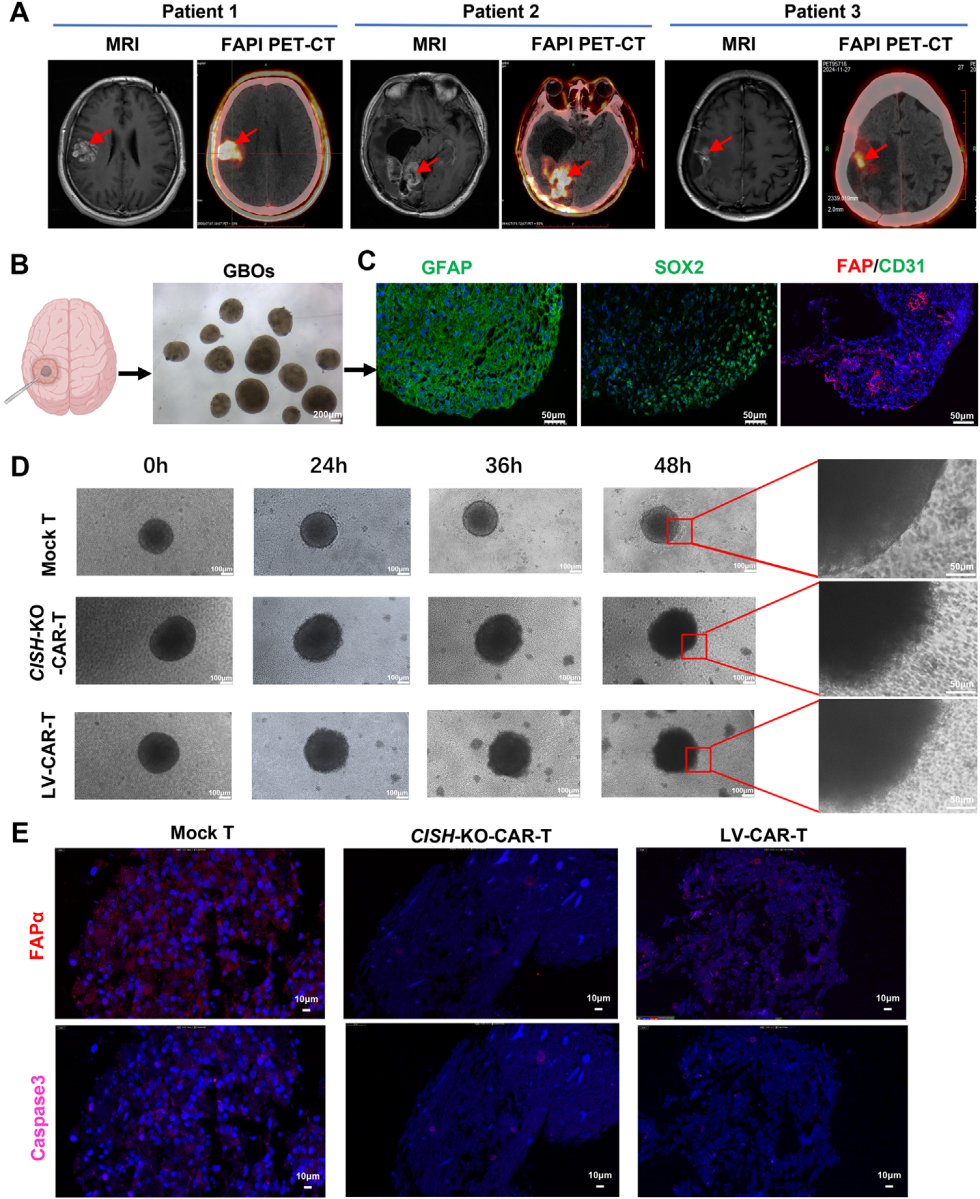
*CISH* locus‐specific integrated FAPα CAR‐T cells effectively kill primary human GBM cells in a patient‐derived glioblastoma organoid model. (A) Three patients are diagnosed with glioblastoma according to their MRI imaging features, including one newly diagnosed and two recurrent GBM patients. The 68Ga‐FAPI‐46 PET‐CT scans that are used to determine FAPα expression in solid tumors are performed in these patients after the informed consent documents are signed. (B) GBOs are generated by culturing micro‐dissected tumor pieces in an optimized medium on an orbital shaker. (C) The expression of GFAP (a traditional astrocyte marker), Sox2 (a marker for GBM stem cells), and FAPα is detected in GBOs by immunofluorescence staining. (D) GBOs are co‐cultured with 1 × 10^5^ FAPα‐targeting CAR‐T cells. After 24 h of co‐culture, the integrity of the organoids cultured with FAPα‐targeting *CISH*‐KO‐ or LV‐CAR‐T cells is significantly disrupted compared to that incubated with mock T cells. (E) GBO immunofluorescence at 72 h post‐treatment shows that GBOs treated with mock T cells exhibit diffuse FAPα expression and a little expression of caspase‐3, a marker of early apoptosis, while GBOs treated with *CISH*‐KO‐ or LV‐CAR‐T cells display massive nuclear debris almost without FAPα and caspase‐3 expression, especially for the *CISH*‐KO‐CAR‐T group.

To determine the capacity of *CISH*‐KO FAPα‐targeting CAR‐T cells in killing primary glioblastoma, we generated patient‐derived glioblastoma organoids (GBOs) from patients undergoing glioblastoma resection surgery. Three patients were diagnosed with glioblastoma according to their magnetic resonance imaging (MRI) features, including one newly diagnosed and two recurrent GBM patients (Figure 8A). The 68Ga‐FAPI‐46 PET‐CT scans used to determine FAPα expression in solid tumors were performed on these patients after they signed the informed consent documents. The results showed the enhanced FAPI uptake in lesions indicated by previous MRI images (Figure 8A), supporting FAPα expression in GBM. GBOs were generated by culturing micro‐dissected tumor pieces in an optimized medium on an orbital shaker (Figure 8B). The expression of GFAP (a traditional astrocyte marker), Sox2 (a marker for GBM stem cells), and FAPα was detected in GBOs by immunofluorescence staining, and we found that GFAP and Sox2 were diffusely expressed in GBOs, indicating the successful establishment of GBOs. FAPα expression was clearly detectable in GBOs, although levels varied (Figure 8C). Next, GBOs were co‐cultured with 1 × 10^5^ FAPα‐targeting CAR‐T cells. After 24 h of co‐culture, the integrity of the organoids cultured with FAPα‐targeting *CISH*‐KO‐ or LV‐CAR‐T cells was significantly disrupted compared to that incubated with mock T cells (Figure 8D). The immunofluorescence staining of GBOs at 72 h post‐treatment showed that GBOs treated with mock T cells exhibited diffuse FAPα expression and a little expression of caspase‐3, a marker of early apoptosis, while GBOs treated with *CISH*‐KO‐ or LV‐CAR‐T cells displayed massive nuclear debris almost without FAPα and caspase‐3 expression, especially for the *CISH*‐KO‐CAR‐T group (Figure 8E), indicating cell death. Taken together, these observations demonstrate that our established *CISH*‐KO FAPα‐targeting CAR‐T cells can effectively kill FAPα‐positive primary GBM cells.

## 3 Discussion

GBM is a highly aggressive and lethal malignant tumor. Currently, clinically meaningful treatment options for relapsed disease after first‐line standard‐of‐care treatment are extremely limited. More recently, CAR‐T cells have been used to treat patients with GBM by targeting GBM‐specific and associated antigens. However, the efficacy of clinical trials of these CAR‐T cells remained limited mainly due to tumor heterogeneity [3]. Therefore, the identification of an ideal targeted antigen is a critical factor for improving the therapeutic efficacy of CAR‐T cells against GBM. Here, we showed a dramatic expression of FAPα in GBM, especially in gliosarcoma, but its absence in normal brain tissues. More importantly, FAPα was highly expressed on both tumor cells and perivascular cells in GBM, indicating potential dual roles of FAPα‐targeting CAR‐T cells in killing tumor cells and destroying tumor‐supporting vascular networks. Therefore, the unique expression pattern of FAPα in GBM makes it a highly attractive target for CAR‐T cell therapy against GBM.

Screening a specific antibody targeting FAPα is crucial for the safety and efficacy of CAR‐T cell therapy against GBM. Compared with the single‐chain fragment variable (scFv) of a monoclonal antibody, nanobodies, also known as camelid VHHs, have shown various advantages when used as the binding domain of CARs due to their small sizes, high stability, less immunogenicity, and less T‐cell exhaustion [21, 34, 35, 36]. Here, we applied a yeast surface display platform developed from rhFAPα‐immunized alpacas to screen VHHs specific for FAPα. To be noted, CD26 has a highly similar sequence and structure to FAPα and is extensively expressed in normal tissues [29]. The engagement of VHH to CD26 probably results in potential off‐target toxicity that could be lethal for CAR‐T cell therapy. To address this issue, CD26^+^FAPα^−^ Huh‐7 cells were used in negative selection for a panel of VHH proteins and VHH‐CAR‐T cells. Additionally, a panel of six cell lines with differential expression patterns of CD26 and FAPα was used to further identify specific killing of candidate 1A10‐CAR‐T cells against FAPα‐expressing target cells, which contributed to the safety and efficacy of FAPα‐targeting CAR‐T cell therapy against GBM. Notably, despite FAPα’s favorable expression profile in GBM, its expression was reported in some specific physiological processes such as embryonic development and wound healing. We propose that the localized administration can mitigate the on‐target, off‐tumor toxicity of anti‐FAPα CAR‐T cells, which is supported by studies showing that local (intratumoral/intrathecal) injection enhanced tumor infiltration, improved efficacy, and reduced peripheral toxicity of CAR‐T cells [37, 38, 39]. In our study, flow cytometric analysis of peripheral blood from treated mice also showed no detectable T cells (data not shown). Therefore, the localized delivery approach helps circumvent a major concern in CAR‐T therapy, constituting a significant advantage for treating GBM.

The defects of engineering T cells using viral vectors have been concerning due to tumorigenic risk [10, 11, 12], high costs [13], and host‐specific immune responses to virus‐derived DNA [14, 15]. More recently, an efficient non‐viral immune cell engineering technology using cssDNA‐mediated genome integration was developed [20]. This non‐viral approach not only addresses the safety and cost concerns associated with viral vectors but also simplifies the regulatory process due to the absence of viral components, which is a significant consideration for clinical manufacturing and approval. Using this approach, we generated non‐viral *CISH* site‐specific integrated FAPα‐targeting CAR‐T cells. The resulting CAR‐T cells achieved highly efficient knockout of *CISH* and targeted CAR integration. Consistent with previous studies that demonstrated *CISH* was a potent negative regulator of TCR signaling and cytokine‐induced T‐cell proliferation [31, 32], our results showed that non‐viral *CISH*‐KO‐CAR‐T cells exhibited significantly enhanced proliferation ability and killing activity against GBM cells, compared to conventional LV‐CAR‐T cells. It is notable that we did not observe a superior anti‐GBM efficacy of *CISH*‐KO‐CAR‐T cells in a GBM xenograft mouse model compared to LV‐CAR‐T cells, even though prolonged survival time was observed. The underlying mechanism could be the decreased or even lost CAR expression in *CISH*‐KO‐CAR‐T cells due to the lack of human cytokines that induced promoter activity of the *CISH* gene in the mouse model. This possibility was supported by our subsequent experiments in which *CISH*‐KO‐CAR‐IL15‐T cells that co‐expressed human IL‐15 exhibited superior efficacy in GBM xenograft mice compared with control LV‐CAR‐IL15‐T cells, indicating an important role of cytokine‐induced *CISH* promoter activity in maintaining CAR expression levels in vivo.

While our findings are promising, we acknowledge several limitations important for interpreting the results and guiding future work. First, while we utilized patient‐derived organoids and immunodeficient mouse models, these systems cannot fully recapitulate the complex GBM tumor microenvironment in patients—particularly the intact vascular network and a complete immune landscape. This limits our ability to assess the potential dual targeting of tumor cells and associated vasculature by FAPα‐specific CAR‐T cells, an interaction that may be critical in a clinical setting. Second, regarding clinical translation, although the non‐viral, site‐specific integration approach presents a favorable safety profile, the long‐term in vivo safety and potential for off‐target effects warrant further investigation in more advanced models prior to clinical application. Addressing these limitations will be essential for the continued development of this therapeutic strategy.

Overall, our proof‐of‐concept study demonstrates that FAPα‐targeting CAR‐T cells engineered by non‐viral *CISH* locus‐specific integration are a practicable and promising therapeutic approach for the treatment of GBM.

## 4 Conclusions

FAPα is an attractive target of CAR‐T cells against GBM due to its dual expression pattern and correlation with poor prognosis. We developed *CISH*‐KO FAPα‐targeting CAR‐T cells via non‐viral cssDNA/CRISPR/Cas9‐mediated CAR gene integration at the *CISH* locus. In preclinical models, these CAR‐T cells exhibited superior anti‐GBM ability compared to conventional LV‐CAR‐T control. This platform offers a mechanistically distinct approach to overcome CAR‐T resistance in GBM, highlighting its strong clinical translational potential for GBM treatment.

## 5 Materials and Methods

### 5.1 Cell Line Culture

293T (catalog number: GNhu17) and 293F (catalog number: SCSP‐5500) cell lines were purchased from the Cell Bank of Chinese Academy of Sciences (Shanghai, China). U87 (catalog number: CL‐0238), LN229 (catalog number: CL‐0578), LX‐2 (catalog number: CL‐0560), Huh‐7 (catalog number: CL‐0120), and SK‐Hep‐1 (catalog number: CL‐0212) cell lines were purchased from Procell Life Science & Technology (Wuhan, China). Jurkat (catalog number: TCH‐C225) cell line was purchased from Haixing Biosciences (Suzhou, China). HEB cell line was purchased from Fenghui Biotechnology (Hunan, China). 293T, U87, LN229, SK‐Hep‐1, and HEB were cultured in DMEM medium (Gibco) supplemented with 10% fetal bovine serum (FBS, Excell Bio). LX2 and Jurkat cell lines were maintained in RPMI‐1640 (Gibco) complete medium supplemented with 10% FBS. 293F cells were maintained in Expi293F Complete Culture Medium (the Cell Bank of the Chinese Academy of Sciences). All cell lines were cultured in media with 1% vol/vol penicillin‐streptomycin solution (Gibco) at a humidified atmosphere of 5% CO_2_ at 37°C. Short tandem repeat (STR) profiling was examined in all cell lines.

### 5.2 Nanobody Selection Using a Yeast Display Library

Nanobody yeast selections were performed as previously described [40] with some modifications. Briefly, a yeast display nanobody library was developed by using peripheral blood mononuclear cells (PBMCs) from a rhFAPα protein‐immunized alpaca. Nanobodies that recognize rhFAPα were isolated from the established yeast display nanobody library using MACS followed by FACS (BD FACSAria III Cell Sorter) selection. After MACS and subsequent FACS for positive selection, a pool of yeast cells that could bind to rh‐FAPα protein was selected. The selected yeast cells were seeded on an SDCAA plate and incubated at 30°C for more than 2 days until they formed visible colonies. A total of 117 single yeast clones were randomly picked, and their bindings to biotin‐labeled FAPα protein were confirmed by flow cytometry. DNA was extracted from each positive‐binding yeast clone. VHH fragments were obtained by PCR using the following primers: 5′‐AGTAA CGTTTGTCAGTAATTGC‐3′ (forward) and 5′‐AGGGTTAGGGATAGGCTTACC TTCG‐3′ (reverse). The VHH sequence for each clone was determined by sequencing the PCR product using the same primers used in PCR.

### 5.3 CAR Vector Design

All CAR expression plasmids share identical components, differing only in the extracellular binding domains due to variations in the nanobody sequences. Nanobody sequences were cloned into the CAR backbone plasmid containing CD8 hinge and transmembrane domain, the 4‐1BB co‐stimulatory domain, the CD3ζ signaling domain, and self‐cleavage P2A followed by EGFP (P2A–EGFP).

### 5.4 Generation of Primary CAR‐T Cells by Lentiviral‐Mediated Gene Transduction

Lentivirus was generated according to the previously described methods [30]. Human primary CD3^+^ T cells were isolated from healthy donor peripheral blood mononuclear cells using a T‐cell isolation kit (STEMCELL Technologies). The CD3^+^ T cells were activated with T Cell TransAct (Miltenyi Biotec) and cultured in TexMACS Medium (Miltenyi Biotec) supplemented with 3% fetal bovine serum and 50 IU/mL human IL‐2 (PeproTech). After activation for 2 days, T cells were transduced with lentivirus (multiplicity of infection = 10) by horizontal centrifugation for 60 min at 32°C and 1000 × *g*. Three days later, the transduced T cells were analyzed for CAR expression by staining with biotinylated FAPα antigen and subsequent streptavidin‐APC (BioLegend) and detecting by flow cytometry. CAR‐T cells were then expanded in TexMACS Medium (Miltenyi Biotec), added with 5 ng/mL recombinant human IL (rhIL)‐15 (PeproTech), 10 ng/mL rhIL‐7 (PeproTech), 3% FBS, and 1% vol/vol penicillin‐streptomycin solution (Gibco) for 10 days before use. CAR‐T cells used in all experiments were normalized to the same positive rates.

### 5.5 Generation of HDR Template Circular Single‐Stranded DNA

Production of cssDNA HDR donor templates was described previously [20]. Specifically, FAPa VHH donor template sequences targeting the *CISH* locus were constructed as double‐stranded DNA (dsDNA) and cloned into a phagemid vector. The M13 helper plasmid and phagemid containing donor template were co‐transformed into an XL1‐Blue *Escherichia coli* strain, and the double‐positive colonies were selected on agar plates with kanamycin (50 µg/mL) and carbenicillin (100 µg/mL). A single colony was picked and grown for approximately 24 h (37°C, 225 rpm) in 250 mL 2×YT media (1.6% tryptone, 1% yeast extract, 0.25% NaCl) to reach OD_600_ between 2.5 and 3.0. The bacteria were pelleted by centrifugation, and the phage particles in the supernatant were precipitated with PEG‐8000. The precipitated phage particles were then pelleted by centrifugation, washed, and lysed in 20 mM MOPS 1 M Guanidine‐HCl, and 2% Triton X‐100. The cssDNA was then extracted from phage lysate with NucleoBond Xtra Midi EF kit (Macherey‐Nagel) following the manufacturer's instructions. Recombinant cssDNA was verified by a nanopore sequencing approach.

### 5.6 Human Primary T‐Cell Electroporation

For preparing non‐viral, gene‐specific targeted CAR‐T cells, primary T cells isolated from healthy donors were stimulated for 2–3 days, and then electroporated using a Lonza 4D electroporation system following the manufacturer's instructions.

In brief, RNPs were prepared by mixing recombinant Cas9 protein (Integrated DNA Technologies, Cat#1081061) with synthetic sgRNA targeting the *CISH* locus (Integrated DNA Technologies), followed by mixture with the cssDNA template (2 µg). Two million T cells were resuspended in 21 µL electroporation buffer P3 (Lonza, Cat# V4XP‐3032) and combined with the RNP/cssDNA mixture to make a total electroporation volume of 25 µL. Program EO115 was chosen for electroporation. After electroporation, cells were immediately supplemented with prewarmed medium and transferred out of the electroporation cuvettes.

### 5.7 CAR‐T In Vitro Cytotoxicity Assays

Cytotoxicity assays were conducted by mixing luciferase‐expressing target cells with CAR‐T cells for 16 h at indicative ratios for each experiment. For the cytotoxicity assay, 150 µg/mL of d‐luciferin (LUCK‐1G, Gold Biotechnology) was added to each sample and incubated for 1 min at room temperature. Luciferase units were measured using a SpectraMax I3X (CLARIO star). Percentages of viable cells were normalized to the bioluminescence of target cells incubated with untransduced T or Mock‐T cells (100% viable), specific lysis was calculated using the formula: specific lysis (%) = 1 − percentage of viable cells, and experiments were performed with technical triplicates.

### 5.8 RNA Sequencing and Analysis

Total RNA was extracted from 5 × 10^6^ cells collected from each group (MOCK‐T, LV‐CAR‐T, and CISH‐KO‐CAR‐T) using Trizol reagent (Invitrogen) following the manufacturer's instructions. RNA integrity and quality were verified by agarose gel electrophoresis and assessed using an Agilent 2100 Bioanalyzer (Agilent Technologies). For library preparation, eukaryotic mRNA was first enriched with Oligo(dT) beads, then fragmented and reverse‐transcribed into cDNA using the NEBNext Ultra RNA Library Prep Kit for Illumina (NEB #7530). The resulting double‐stranded cDNA fragments underwent end repair, adenylation, and Illumina adapter ligation. After purification with AMPure XP Beads (1.0X) and PCR amplification, the constructed libraries were subjected to paired‐end sequencing on an Illumina Novaseq 6000 platform at Gene Denovo Biotechnology Co. (Guangzhou, China).

### 5.9 Murine Experiments

Four‐ to six‐week‐old female NCG (NOD/ShiLtJGpt‐Prkdc^em26Cd52^IL2rg^em26Cd22^/Gpt) mice were purchased from GemPharmatech Co. Tumor cells were implanted intracranially as previously described [41]. Briefly, a total of 3 × 10^5^ U87‐luc cells in 5 µL HBSS were injected into the right forebrain, using a stereotactic device. Tumor formation was monitored using in vivo bioluminescence imaging (BLI). After confirmation of engraftment 7 days post‐injection (Day 0), mice were given a single injection of RNP(*CISH*‐KO)‐T *CISH*‐KO‐CAR‐T, or LV‐CAR‐T cells (1 × 10^6^ cells per mouse) by orthotopic injection at tumor sites. Subsequently, BLI was used to monitor tumor luminescence intensity in order to assess tumor growth, and bodyweight was recorded. All animal experiments adhered to the guidelines for the care and use of laboratory animals and were approved by the Ethics Committee of the Animal Experiment Center of Nanfang Hospital, Southern Medical University (IACUC‐LAC‐20240401‐004).

### 5.10 Preparation of Single‐Cell Suspension From Mouse Tumor Tissue

Mouse tumor tissue was processed in a culture dish. The tumor was minced into small fragments using a scalpel, followed by the addition of tumor digestion solution (STEMCELL). The mixture was incubated at 37°C for 25 min. Digestion was then stopped, and the cell suspension was filtered through a 70 µm cell strainer. The filtrate was centrifuged at 300 × *g* for 10 min at room temperature. The pellet was collected and treated with red blood cell lysis buffer (Solarbio) according to the manufacturer's instructions. Finally, the cells were harvested for subsequent flow cytometry analysis.

### 5.11 Flow Cytometry

Flow cytometry, 3 × 10^5^ cells, unless otherwise noted, in 100 µL total volume of FACS buffer (PBS +2% FBS) were stained using the manufacturer's recommended amount of antibody for 30 min, and then washed with excess FACS buffer to remove unbound antibody. Samples were immediately analyzed using either a CytoFLEX Flow Cytometer (Beckman Coulter) or a FACSAria II flow cytometer (BD Biosciences). Antibodies used for flow cytometry are listed in Table S1.

### 5.12 Immunohistochemistry

Paraffin sections or patient tissues were obtained from the Department of Pathology at Sanjiu Brain Hospital with the approval of the Ethics Committee (NFEC‐202407‐K17). For fresh patient tissues, 4% paraformaldehyde was used for fixation, followed by paraffin embedding, sectioning, and deparaffinization. Antigen retrieval was performed using a microwave (15 min once the boiling point was achieved) in PH 9.0 EDTA buffer solution. After cooling, the sections were washed and treated with 3% hydrogen peroxide to quench endogenous peroxidase activity for 30 min. Then, 10% normal goat serum (#AR1009, Boster) was used to block non‐specific antibody binding. After removing the blocking solution, the sections were incubated overnight at 4°C with anti‐FAPα (#ab207178, Abcam). Following extensive washing, a secondary antibody (#ab205718, Abcam) was used for FAPα staining and incubated with the sections at room temperature for 45 min. After extensive washing, detection was performed using an HRP kit (#p0603, Beyotime) as recommended by the manufacturer, followed by a reaction with a DAB peroxidase substrate kit (#DAB4033, Maxim). The sections were then counterstained with hematoxylin (#C0107, Beyotime) and mounted with a mounting medium.

### 5.13 Immunofluorescence Staining

Paraffin sections were sequentially treated with the dewaxing agent series (YA0031, Solarbio) and an ethanol gradient (absolute ethanol, 95% ethanol, 75% ethanol) to achieve dewaxing to water. Antigen retrieval was performed using a microwave (15 min once the boiling point was achieved) in PH 9.0 EDTA buffer solution. The sections were then blocked with 3% hydrogen peroxide, followed by sealing with 10% goat serum for 30 min, and incubated overnight with a 1:8000 dilution of CD3 (ab237721, Abcam) or CD31(ab182981, Abcam) primary antibody solution. The next day, after equilibration to room temperature, the sections were incubated for 45 min with a 1:4000 dilution of goat anti‐rabbit IgG H&L (HRP) secondary antibody solution (ab205718, Abcam). IFluor 488 tyramide (#45100, AAT Bioquest) working solution was used for TSA staining. Afterwards, the sections were eluted in a 42°C water bath with elution solution for 20 min. Re‐blocking was performed using 10% donkey serum (ANT051, Antgene), followed by overnight incubation with anti‐FAPα (#ab207178, Abcam) primary antibody solution. The next day, the sections were incubated for 45 min with a dilution of Donkey anti‐Rabbit IgG (H+L) Alexa Fluor 594 (A21207, ThermoFisher) secondary antibody solution, followed by a 5‐min nucleus staining with a 1:500 dilution of DAPI (C0060, Solarbio) working solution. Finally, the sections were sealed with a fluorescence mounting medium (#S2100, Solarbio) and stored at 4°C in the dark.

### 5.14 Establishment and Cultivation of Organoids

Tumor tissues were collected from patients with glioblastoma undergoing surgery at Guangdong Sanjiu Brain Hospital with the approval of the Ethics Committee (NFEC‐202407‐K17). These tissues were stored in live tissue preservation medium (Mogengel Biotechnology) and placed on ice for rapid transportation to the laboratory. Upon arrival, each tissue sample was transferred to a sterile dish containing precooled DPBS (Gibco) with 1% penicillin‐streptomycin (Gibco), under a stereomicroscope. The tissues were rinsed three times and then minced into approximately 0.5–1 mm diameter pieces using sterile dissection scissors. After mincing, the tissue pieces were washed three more times to remove cellular debris. Pieces containing substantial amounts of necrosis or surrounding brain tissue were discarded. The remaining tumor pieces were incubated in 1X RBC lysis buffer (#00‐4333‐57, Invitrogen) under gentle rotation for 10 min at room temperature to lyse the majority of contaminating red blood cells. The RBC lysis buffer was then aspirated, and the tumor pieces were washed three times in DMEM:F12 medium (Gibco). Finally, the supernatant was aspirated, and the glioma pieces were placed into human glioma culture medium in an ultra‐low attachment six‐well plate (Corning).

### 5.15 Statistics

Experimental data are presented as the mean ± SE as described in the figure legends.

Two independent groups were compared using a two‐tailed *t*‐test. Tumor growth in vivo was compared using two‐way repeated‐measures analysis of variance. Survival patterns in tumor‐bearing mice were analyzed by the Kaplan–Meier method, and statistical differences were assessed according to the Mantel–Cox log‐rank test. *p* < 0.05 was considered to be statistically significant. All of the statistical analyses were conducted using SPSS Statistics 22 software. Unless otherwise stated, graphs were created using GraphPad Prism version 8.00 (GraphPad Software; www.graphpad.com). Asterisks are used to indicate significance: *p < 0.05, **p < 0.01, ***p < 0.001, ****p < 0.0001; NS, no significance.

## Author Contributions

X.D. performed the main experiments, analyzed the results, produced the figures, and wrote the manuscript. Y.S. and T.‐Y.G. performed partial experiments. J.W. contributed to sgRNA design and cssDNA. F.W., Z.‐M.W., R.‐Z.L., T.‐T.T., S.Z., and X.‐T.L. contributed to in vitro experiments. F.X. and H.‐B.W. were responsible for the PET‐CT examination. Z.‐Z.G. and R.‐H.H. contributed to in vivo experiments. H.W. designed the experiments and contributed to the manuscript. X.‐B.Z. and B.‐J.C. designed the experiments and the pathological experiment. G.‐Z.X. conceptualized and designed this study, provided administrative, technical, and material support, prepared figures, and wrote the manuscript. All authors read and approved the final version of the manuscript.

## Funding

This work was supported by the Hospital‐Industry Collaboration Project (GRGZ2022330 and GRGZ20230520), the National Natural Science Foundation of China grants (82373302), the Science and Technology Projects of Guangzhou (2024A04J6613), and the Science and Technology Plan Project of Guangdong (2024A0505040015).

## Ethics Statement

This study was approved by the Medical Ethics Committee of Nanfang Hospital of Southern Medical University (Ethics Approval Number: NFEC‐202407‐K17). All animal experiments adhered to the guidelines for the care and use of laboratory animals and were approved by the Ethics Committee of the Animal Experiment Center of Nanfang Hospital, Southern Medical University (IACUC‐LAC‐20240401‐004). Patient‐related data were obtained with written informed consent from all participants.

## Conflicts of Interest

Authors Jiao Wang and Hao Wu are employees of Full Circles Therapeutics, but have no potential relevant financial or non‐financial interests to disclose. The remaining authors declare no conflicts of interest.

## Supporting information




**Supporting Figure 1**: FAPα‐binding yeast clones identified by flow cytometry. Total 117 single yeast clones were randomly picked, and their bindings to biotinylated FAPα protein were confirmed by flow cytometry.
**Supporting Figure 2**: The binding of VHH‐Fc fusion protein to target cells. VHH‐Fc fusion proteins are produced in a mammalian expression system, and incubated with wild‐type (wt‐293T, upper panels) or FAPα‐overexpressing 293T cells (FAPα‐293T, lower panels). Binding is assessed by flow cytometry, demonstrating specific enrichment on FAPα‐293T cells compared to the isotype control and wt‐293T cells.
**Supporting Table 1**: Antibodies used for flow cytometry.

## Data Availability

The data that support the findings of this study are available from the corresponding author upon reasonable request. The bulk RNA‐seq data have been deposited in Gene Expression Omnibus (GEO) with GEO accession GSE314496.
