# Supplementary material for: Non‐Viral Cytokine‐Inducible SH2 Containing Protein Locus‐Specific Integrated Fibroblast Activation Protein Alpha‐Targeting Chimeric Antigen Receptor T Cells Achieve Potent Antitumor Efficacy in Glioblastoma
Source: MedComm (2020). 2026 Mar 26;7(4):e70702. doi: 10.1002/mco2.70702 (PMC13042763; doi:10.1002/mco2.70702)
Supplement: Supplementary file 1 — Supporting Figure 1: FAPα‐binding yeast clones identified by flow cytometry. Total 117 single yeast clones were randomly picked, and their bindings to biotinylated FAPα protein were confirmed by flow cytometry. Supporting Figure 2: The binding of VHH‐Fc fusion protein to target cells. VHH‐Fc fusion proteins are produced in a mammalian expression system, and incubated with wild‐type (wt‐293T, upper panels) or FAPα‐overexpressing 293T cells (FAPα‐293T, lower panels). Binding is assessed by flow cytometry, demonstrating specific enrichment on FAPα‐293T cells compared to the isotype control and wt‐293T cells. Supporting Table 1: Antibodies used for flow cytometry. [file MCO2-7-e70702-s001.docx]

**Non-viral** cytokine inducible SH2 containing protein **locus-specific integrated** fibroblast activation protein alpha-targeting chimeric antigen receptor T **cells achieve potent anti-tumor efficacy in glioblastoma**

Xin Dong^1#^, Yao Sun^1#^, Yuetong Guo^1#^, Jiao Wang^2#^, Fei Wang^1^, Ziming Wang^1^, Ruizhen Li^1^, Fei Xie^3^, Tingting Tan^1^, Baijie Cheng^4^, Ronghan Huang^1^, Shu Zhang^1^, Xiaotong Lin^1^, Zhaoze Guo^5^, Hubing Wu^4^, Hao Wu^2*^, Xubiao Zhang^6*^, Guozhu Xie^1,7*^

^1^ Department of Radiation Oncology, Nanfang Hospital, Southern Medical University, Guangzhou, Guangdong Province, China.

^2^ Full Circles Therapeutics, Cambridge, MA, USA.

^3^ Nanfang PET center, Nanfang Hospital, Southern Medical University, Guangzhou, Guangdong Province, China.

^4^ Department of Pathology, Guangdong Sanjiu Brain Hospital, Guangzhou, Guangdong Province, China.

^5^ Breast Center, Department of General Surgery, Nanfang Hospital, Southern Medical University, Guangzhou, Guangdong Province, China.

^6^ Department of Neurosurgery, Guangdong Sanjiu Brain Hospital, Guangzhou, China.

^7^ Guangdong Provincial Key Laboratory of Viral Hepatitis Research, Guangzhou, Guangdong Province, China.

**^#^Co-first authors.**

**^*^Correspondence to:** Guozhu Xie, E-mail: [xieguozhu@126.com;](mailto:xieguozhu@126.com;) Xubiao Zhang, zhangxubiao999@163.com; Hao Wu, [howard.wu@fullcirclestx.com.](mailto:howard.wu@fullcirclestx.com;)

**Supplemental Methods**

**IL-15 ELISA**

To quantify IL-15 in the supernatant of CAR-T cell cultures, an ELISA assay was conducted using a Human IL-15 ELISA Kit ( NeoBioscience EHC013.96 ). Initially, normalized the positivity rate of all CAR-T cells to 15% using untransduced T cells, then co-culture them at a 1:1 E:T ratio with U87-MG cells or without target cells for 48 hours. Supernatants were collected from CAR-T cell cultures post-incubation and stored at -80°C until downstream analysis. Proceeded with the subsequent testing steps according to the manufacturer's protocol.

**Public data origination**

Gene expression and clinical data were acquired from public repositories. The TCGA-glioma dataset was downloaded from the Cancer Genome Atlas (TCGA) in August 2024, including gene expression profiles of 705 glioma samples and 5 adjacent normal samples, as well as clinical information for 682 patients. Additionally, one dataset was obtained from the Gene Expression Omnibus under the accession numbers GSE50161 (117 gliomas of diverse histologies vs. 13 controls).

**Bioinformatics analysis**

The preprocessing of raw data from GEO was performed using the ‘limma’ package (Version 3.58.1) of the R software (Version 4.3.3 ) [[1](https://cancerci.biomedcentral.com/articles/10.1186/s12935-020-01515-1" \l "ref-CR9" \o "Ritchie ME, Phipson B, Wu D, Hu Y, Law CW, Shi W, et al. Limma powers differential expression analyses for RNA-sequencing and microarray studies. Nucleic Acids Res. 2015;43(7):e47.)], and then data normalization of samples from each expression profiles was conducted by using robust multi-array average (RMA) method [2, 3], including background adjustment, quantile normalization, and log 2 conversion. Afterwards, the probes were annotated with the platform annotation file.

Differential expression analysis was performed using the ‘edgeR’ package (Version 4.0.16) [4]. The expression data from TCGA were presented in the form of transcripts per million(TPM) and normalized to the library size using the trimmed mean of M-values (TMM) method, which calculated counts per million (CPM), considering normalization coefficients.

**Survival analysis**

The samples with no overall survival (OS) time (or less than one mouth) were removed. Afterwards, the remaining samples were divided into high expression group and low expression group based on the median expression levels of genes. Survival analysis was performed using Kaplan-Meier and the log-rank statistical test P < 0.05 was regarded as statistically significant threshold.

***CISH* Locus Deletion/Insertion Validation by PCR**

**Sample preparation**

5×10^6^ cells from Mock-T, RNP-T, and *CISH*-KO-CAR-T groups were collected. Genomic DNA was purified using Genomic DNA Purification Kit (Vazyme DC102) following standard protocols.

**PCR amplification**

Target regions containing indels were amplified with gene-specific primers. Separate PCR conditions were optimized for deletion vs. insertion validation:

1. CISH Deletion PCR (35 cycles):
   - Annealing: 64°C (25s)
   - Extension: 12s (optimized for short indel detection)
2. CAR Insertion PCR (35 cycles):
   - Annealing: 64°C (25s)
   - Extension: 46s (accounts for longer CAR insert size)

**Purification and sequencing**

PCR products were purified using Gel DNA Purification Kit (Vazyme DC301). Sanger sequencing was conducted to confirm indel frequencies, and the indel percentage was measured by Inference of CRISPR Edits (ICE) analysis (Synthego). The primers used were as follows: CISH-Forward: 5′-CCGACAACACCTGCAGAAGAT-3′; CISH-Reverse: 5′-GGTACTGTCGGAGGTAGT-3′.

**Supplemental Figures**

**Figure S1**


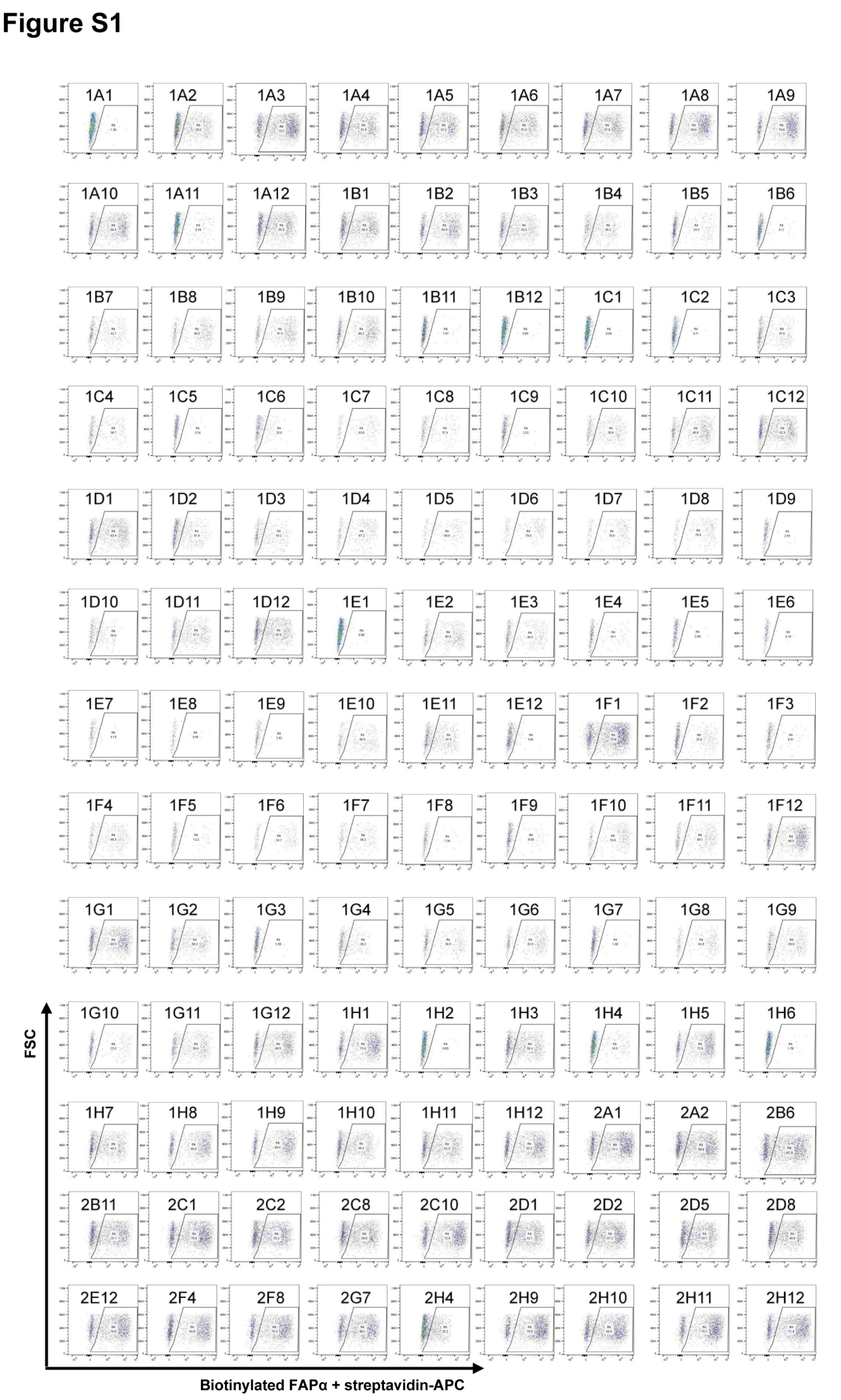


**Figure S1 FAPα-Binding Yeast Clones Identified by Flow Cytometry.** One hundred and seventeen single yeast clones were randomly picked, their bindings to biotinylated FAPα protein were confirmed by flow cytometry.

**Figure S2**

**
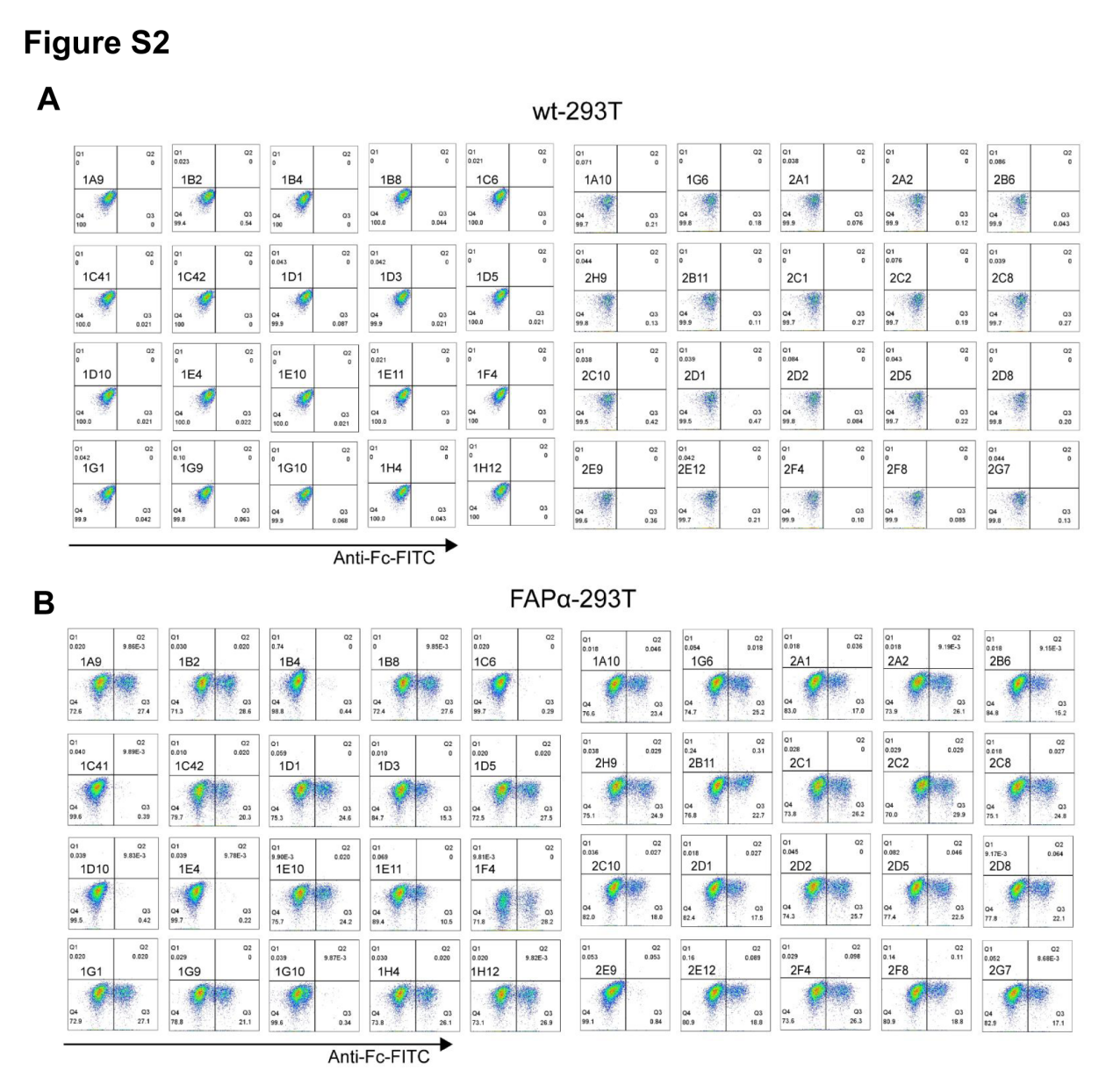
**

**Figure S2 The binding of VHH-Fc fusion protein to target cells.** VHH-Fc fusion proteins were produced in a mammalian expression system, and incubated with wild-type (wt-293T, upper panels) or FAPα-overexpressing 293T cells (FAPα-293T, lower panels). Binding was assessed by flow cytometry, demonstrating specific enrichment on FAPα-293T cells compared to the isotype control and wt-293T cells.

**Supplemental Table 1**

**Table S1 Antibodies used for flow cytometry.**

| **Antibody Name** | **Manufacturer** | **Cat.** |
| --- | --- | --- |
| PE/Cyanine7 anti-human TIGIT(VSTM3) | Biolegend | 372713 |
| Brilliant Violet 421TM anti-human CD279(PD-1) | Biolegend | 329920 |
| Brilliant Violet 605^TM^ anti-human CD366(Tim3) | Biolegend | 345017 |
| PE anti-human CD366(Tim-3) | Biolegend | 345006 |
| APC Streptavidin | Biolegend | 405207 |
| PE anti-human CD3 | Biolegend | 980008 |
| anti-hFAP Alxa Fluor 647 Conjugated | R&D Systems | FAB3715R |

**References**

[1]Ritchie ME, Phipson B, Wu D, Hu Y, Law CW, Shi W, et al. Limma powers differential expression analyses for RNA-sequencing and microarray studies. Nucleic Acids Res. 2015;43(7):e47. doi: 10.1093/nar/gkv007

[2] Bolstad BM, Irizarry RA, Åstrand M, Speed TP. A comparison of normalization methods for high density oligonucleotide array data based on variance and bias. Bioinformatics. 2003;19(2):185–193. doi: 10.1093/bioinformatics/19.2.185

[3] Irizarry RA, Hobbs B, Collin F, Beazer-Barclay YD, Antonellis KJ, Scherf U, et al. Exploration, normalization, and summaries of high density oligonucleotide array probe level data. Biostatistics. 2003;4(2):249–264. doi: 10.1093/biostatistics/4.2.249

[4] Robinson M. D., McCarthy D. J., Smyth G. K. edgeR: a Bioconductor package for differential expression analysis of digital gene expression data. Bioinformatics. 2010;26 (1), 139–140. doi: 10.1093/bioinformatics/btp616
